# Whole-Genome Sequencing Analysis of *Salmonella* *Enterica* Serotype Enteritidis Isolated from Poultry Sources in South Korea, 2010–2017

**DOI:** 10.3390/pathogens10010045

**Published:** 2021-01-07

**Authors:** Ji-Yeon Hyeon, Shaoting Li, David A. Mann, Shaokang Zhang, Kyu-Jik Kim, Dong-Hun Lee, Xiangyu Deng, Chang-Seon Song

**Affiliations:** 1 Center for Food Safety, Department of Food Science and Technology, The University of Georgia, 1109 Experiment St, Griffin, GA 30223, USA; jiyeon.hyeon@uconn.edu (J.-Y.H.); shaoting.li25@uga.edu (S.L.); dmann29@uga.edu (D.A.M.); zskzsk@uga.edu (S.Z.); xdeng@uga.edu (X.D.); 2 Department of Pathobiology and Veterinary Science, The University of Connecticut, 61 N. Eagleville road, Storrs, CT 06269, USA; dong-hun.lee@uconn.edu; 3 KCAV Inc, KU Future Energy Research Center 202, 120 Neungdong-ro, Gwangjin-gu, Seoul 05029, Korea; zidanov31@gmail.com; 4 Avian Disease Laboratory, College of Veterinary Medicine, Konkuk University, 120 Neungdong-ro, Gwangjin-gu, Seoul 05029, Korea

**Keywords:** *Salmonella* enteritidis, poultry, antibiotic resistance gene, virulence gene, SNP analysis, cgMLST analysis

## Abstract

*Salmonella enterica* subsp. *enterica* serotype Enteritidis (SE) is recognized as a major cause of human salmonellosis worldwide, and most human salmonellosis is due to the consumption of contaminated poultry meats and poultry byproducts. Whole-genome sequencing (data were obtained from 96 SE isolates from poultry sources, including an integrated broiler supply chain, farms, slaughterhouses, chicken transporting trucks, and retail chicken meats in South Korea during 2010–2017. Antimicrobial resistance and virulence genes were investigated using WGS data, and the phylogenetic relationship of the isolates was analyzed using single-nucleotide polymorphism (SNP) typing and core genome multilocus sequence typing (cgMLST). All isolates carried aminoglycoside resistance genes, *aac(6’)-Iaa*, and 56 isolates carried multiple antimicrobial resistance genes. The most frequent virulence gene profile, *pef-fim-sop-inv.-org-sip-spa-sif-fli-flg-hil-ssa-sse-prg-pag-spv*, was found in 90 isolates. The SNP analysis provided a higher resolution than the cgMLST analysis, but the cgMLST analysis was highly congruent with the SNP analysis. The phylogenetic results suggested the presence of resident SE within the facility of processing plants, environments of slaughterhouses, and the integrated broiler supply chain, and the phylogenetically related isolates were found in retail meats. In addition, the SE isolates from different origins showed close genetic relationships indicating that these strains may have originated from a common source. This study could be valuable reference data for future traceback investigations in South Korea.

## 1. Introduction

Nontyphoidal *Salmonella enterica* (*S. enterica*) is one of the most prevalent foodborne pathogens in the world [1,2]. Among *Salmonella* serotypes, *S. enterica* serotype Enteritidis (*S*. Enteritidis, SE) is the most common *Salmonella* serotype associated with foodborne illnesses in the United States and Europe, and its common sources are poultry and poultry products [3]. In South Korea, *S. enterica* was the second most common pathogen associated with foodborne illnesses over the past 15 years, according to the foodborne outbreak surveillance system of Korea’s Food and Drug Administration (https://www.foodsafetykorea.go.kr/portal/healthyfoodlife/foodPoisoningStat.do). To identify infection sources and transmission routes, the epidemiological association between poultry sources and human disease has been investigated using different molecular typing methods [4,5,6,7].

Recently, whole-genome sequencing (WGS) has been frequently implemented in routine surveillance of foodborne pathogens as well as foodborne disease outbreak investigation [2,7,8,9,10,11]. WGS enables serotyping, antimicrobial resistance, virulence profiling, and subtyping in a single WGS workflow [12]. In addition, the application of WGS in laboratory surveillance provides higher resolution and precision than pulsed-field gel electrophoresis (PFGE), which had been the gold standard for PulseNet [12]. Therefore, PulseNet has worked toward implementing WGS into routine public health surveillance since 2014, and PFGE has been phased out and replaced with WGS as PulseNet’s primary surveillance tool in 2019 [13]. Currently, there are several studies that applied WGS to molecular characterization and phylogenetic analysis of the *Salmonella* spp. isolated from various sources [2,5,7,14,15,16,17,18,19,20]. However, only a few studies using WGS analysis for *Salmonella* spp. in South Korea have been reported [21,22,23], and WGS is not yet routinely used as a tool for genetic characterization of SE in the food supply chain in South Korea.

There are two analytical approaches of WGS-based subtyping: base-by-base (single-nucleotide polymorphism [SNP] analysis) or gene-to-gene (multilocus sequence typing [MLST]) [24]. For the base-by-base approach, an SNP is a nucleotide difference at a specific position in the genome of a test strain relative to the sequence of a reference strain and occurs because of a genetic mutation event [24]. The MLST approach considers allelic variants in a defined set of gene loci [19]. This approach is very flexible because the number and the nature of the genes assessed can be tailored to any given situation and genomes in question [24]. Core genome MLST (cgMLST) balances the number of loci used in a scheme by including those loci present in the majority of isolates (ranging from 95% to 99%) in a given species [17].

Given the significance and repeated outbreaks of SE in South Korea together with the limited availability of WGS data, we sequenced the whole genome of 96 SE isolates isolated from poultry sources in South Korea during 2010–2017, including retail chicken meats, farms, slaughterhouses, an integrated broiler supply chain, and chicken transporting trucks. The presence of antimicrobial resistance genes, chromosomal mutation, and virulence genes in the SE isolates was investigated using WGS data. Then we conducted a phylogenetic analysis using SNP and cgMLST analysis to investigate the genetic relatedness of SE isolates sequenced in this study.

## 2. Results

### 2.1. Detection of Antimicrobial Resistance Genes and Mutations in the gyrA, gyrB, parC and parE Genes

**Figure 1:**
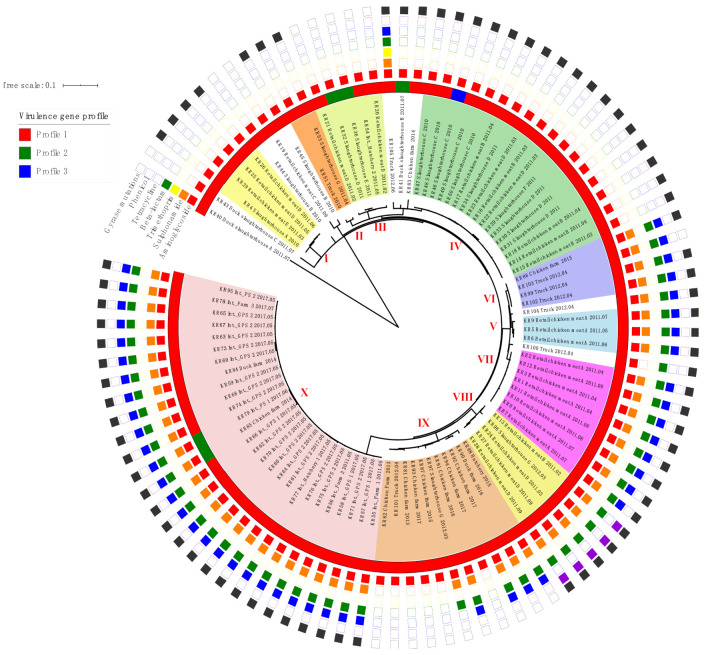
Phylogenetic single-nucleotide polymorphism (SNP) tree, antibiotic resistance patterns and virulence gene profiles of 96 SE isolates visualized using interactive Tree Of Life version 5 (iTOLv5) (https://itol.embl.de/). The SE isolates belonging to SNP groups (I to X) are highlighted with different colors, and the presence of antimicrobial genes and virulence gene profiles of each SE isolates are indicated. Int., integrated broiler supply chain; GPS, grandparent stock; PS, parent stock.

The WGS data were used to detect the presence of antimicrobial resistance genes in the 96 isolates, and a total of 12 antimicrobial resistance patterns were observed (Table 1 and Supplementary Table S1). The most frequent antimicrobial resistance pattern was pattern 1 (*n* = 27, 28.1%), followed by pattern 10 (*n* = 19, 19.8%) and pattern 5 (*n* = 15, 15.6%). Among 96 isolates, 56 isolates (58.3%) carried multiple antimicrobial resistance genes (Table 1), and one isolate from the chicken transporting truck (KR105) contained 5 antimicrobial resistance genes (Figure 1). A total of 12 antibiotic resistance genes were identified and are described in detail below, according to the different antimicrobial classes (Table 1).

#### 2.1.1. Aminoglycoside Resistance Genes

All 96 isolates carried aminoglycoside resistance genes, *aac(6’)-Iaa*, and four other different genes including *aph(6)-Id* (*n* = 53, 55.2%), *aph(3")-Ib* (*n* = 53, 55.2%), *aac(3)-Iid* (*n* = 17, 17.7%), and *aph(3’)-Ia* (*n* = 16, 16.7%) were also detected.

#### 2.1.2. Sulfonamide and Trimethoprim Resistance Genes

Two sulfonamide resistance genes were found in 55 (57.3%) isolates including *su*l2 (*n* = 54, 56.2%) and *sul1* (*n* = 1, 1.0%), and only one trimethoprim resistance gene, *dfr*A1, was detected in two (2.1%) isolates.

#### 2.1.3. Beta-Lactam Resistance Genes

Two beta-lactams resistance genes were detected in 56 (58.3%) isolates including *bla*_TEM-1B_ (*n* = 39, 40.6%) and *bla*_CTX-M-15_ (*n* = 17, 17.7%).

#### 2.1.4. Tetracycline Resistance Genes

Only one tetracycline resistance gene, *tet*(*A)*, was detected in 48 (50%) isolates.

#### 2.1.5. Phenicol Resistance Genes

One phenicol resistance gene, *catA2*, was found in five (5.2%) isolates.

#### 2.1.6. Detection of Mutations in the *gyrA*, *gyrB*, *parC* and *parE* Genes

A total of 75 (78.1%) out of 96 isolates presented point mutation in the *gyrA* gene, with all being resistant to nalidixic acid and ciprofloxacin (Figure 1, Supplementary Table S1). The nonsynonymous point mutation in the *gyrA* gene included: aspartate/glycine, D87G in 25 isolates (26.0%); aspartate/asparagine, D87N in 50 isolates (52.1%). None of the isolates had point mutation n the *gyrB*, *parC* and *parE* genes (data not shown).

### 2.2. Virulence Gene Profiles

The genomes SE isolates tested were investigated for 21 virulence genes. From the results of the virulence gene investigation, three virulence gene profiles were established (Figure 1, Table 2). The most frequently detected profile was profile 1 (*pef-fim-sop-inv.-org-sip-spa-sif-fli-flg-hil-ssa-sse-prg-pag-spv*) (*n* = 90, 93.8%) followed by profile 2 (*fim-sop-inv.-org-sip-spa-sif-fli-flg-hil-ssa-sse-prg-pag*) (*n* = 5, 5.2%) and profile 3 (*pef-fim-sop-inv.-org-sip-spa-sif-fli-flg-hil-ssa-sse-prg-pag*) (*n* = 1, 1.0%). All isolates carried *fim*, *sop*, *inv*, *org*, *sip*, *spa*, *sif*, *fli*, *flg*, *hil*, *ssa*, *sse*, *prg*, and *pag*, and none of the strains carried *sef*, *sfm*, *spr*, *stn*, and *bss* (Table 2).

### 2.3. Phylogenetic Analysis of SE Isolates

**Figure 2:**
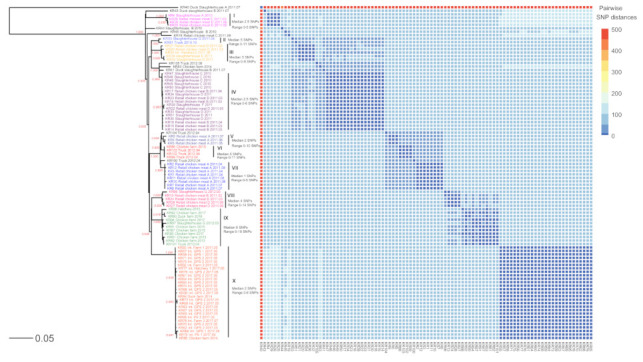
Phylogenetic analysis of 96 SE isolates using SNP analysis. The phylogeny was rooted at the midpoint. A total of 1034 SNP sites were identified. The colored SE isolates were grouped into 10 groups (I to X). Pairwise SNP distances (0 to 492 SNPs) are indicated by the heat map. Colors in the heat map indicate the numbers of pairwise SNP distances between isolates, with blue being the lowest and light orange being the largest. The scale bar measures the number of substitutions per site. Ratios on certain tree branches indicate branch support by the default approximate likelihood ratio test (aLRT) in PhyML. Only support greater than 0.9 is shown. Int., integrated broiler supply chain; GPS, grandparent stock; PS, parent stock.

**Figure 3:**
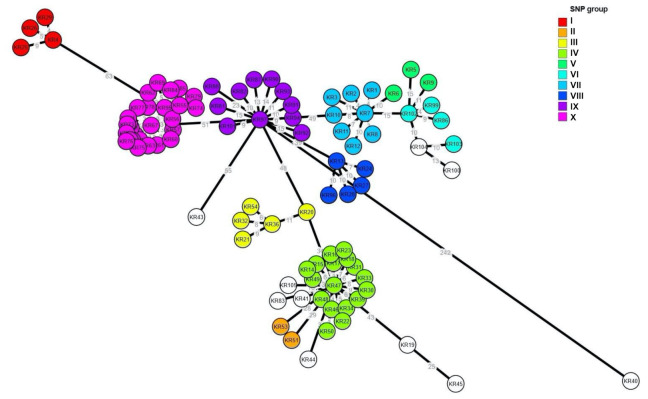
Core genome multilocus sequence typing (cgMLST)-based minimum spanning tree [created with cgMLST Finder 1.1(https://cge.cbs.dtu.dk/services/CSIPhylogeny/) and GrapeTree version 1.5.0 (https://github.com/achtman-lab/GrapeTree)] of the 96 SE isolates of this study. The colors of the nodes indicate the groups of SNP analysis (I to X), and the numbers at the branches indicate the allelic differences to the nearest neighbor.

The 96 SE genomes were assessed for their phylogenetic relationships using both SNP and cgMLST analysis (Figure 1, Figure 2 and Figure 3). According to the framework for interpreting SNP analysis provided by the FDA-CFSAN [25], we considered the isolates are closely related, increasing the likelihood that they arose from the same source when i) there are 20 or fewer SNPs and ii) the phylogenetic analysis shows a monophyletic relationship with bootstrap support of 0.90 or higher. The SE genomes were grouped into 10 different groups (I to X), and ten singleton genomes were not clustered into any major group (Figure 1 and Figure 2). The SE isolates in the identical phylogenetic groups showed similar antibiotic resistance patterns (Figure 1).

In SNP phylogeny (Figure 2), group I consisted of four closely related isolates from the retail chicken meats of brand D (*n* = 2) and E (*n* = 1) and the slaughterhouse A (*n* = 1) (median 2.5 SNPs, range 0–5 SNPs). Two isolates from the slaughterhouse G and the truck clustered in the group II (median 5 SNPs, range 0–11 SNPs). Group III included five isolates from the retail chicken meats of brand D (*n* = 2), slaughterhouse D (*n* = 1) and E (*n* = 1), and the hatchery 2 of the integrated broiler supply chain (*n* = 1) (median 5 SNPs, range 0–9 SNPs). In group IV, five of six isolates from the retail chicken meats of brand B were clustered together with the isolates from the retail chicken meats of brand D (*n* = 2) and the slaughterhouse C (*n* = 5), D (*n* = 4), and F (*n* = 1) (median 2.5 SNPs, range 0–6 SNPs). In group V, three isolates from the retail chicken meats of brand A clustered together (median 2 SNPs, range 0–10 SNPs). Group VI contained four isolates from the trucks (*n* = 3) and a chicken farm (*n* = 1) (median 5 SNPs, range 0–11 SNPs), and eight SE isolates from the retail chicken meats of brand A clustered together in the group VII (median 1 SNPs, range 0–5 SNPs). Group VIII included five isolates from the retail meat chicken meats of brand B (*n* = 1) and D (*n* = 3) and the slaughterhouse G (*n* = 1) (median 4 SNPs, range 0–14 SNPs). In group IX, seven isolates from chicken farms were clustered together with the isolates from the duck farm (*n* = 1), the slaughterhouse G (*n* = 1), and the truck (*n* = 1) (median 8 SNPs, range 0–18 SNPs). Group X included 27 isolates from the integrated broiler supply chain (*n* = 25), duck farm (*n* = 1) and chicken farm (*n* = 1) (median 2 SNPs, range 0–8 SNPs). Among the 26 isolates from the integrated broiler supply chain, 25 isolates were clustered together into group X.

The results of cgMLST analysis showed that the clustering of the isolates resembled that of the SNP analysis, such as the SNP group I, III, VIII, IX and X (Figure 3). However, there were some differences in clustering from the SNP analysis. The SE isolates in the SNP group V, VI and VII were grouped into two clusters, and the SNP group II and IV were clustered together in cgMLST.

## 3. Discussion

WGS analysis has been used to investigate genetic characteristics, and phylogenetic relationships among *Salmonella* isolates from different origins, such as humans, food, animals or environmental samples in previous studies [6,14,17,25]. In this study, the genomes of SE isolates from poultry sources in broiler production chains in South Korea were sequenced to evaluate their antimicrobial resistance gene pattern, virulence gene profile and phylogenetic diversity.

Several previous studies reported that phenotypic resistance and genotypic resistance correlated highly in *Salmonella* spp. [15,17,26,27], but some of the discrepancies in aminoglycosides and beta-lactams were noted [14,15,17,27]. Genotypic antimicrobial resistance found in this study agreed with the phenotypic antimicrobial resistance of *Salmonella* spp. from poultry sources, in that there was high resistance to streptomycin, nalidixic acid, sulfisoxazole, and cephalothin, found in previous studies from South Korea [26,27,28]. The finding of multidrug-resistant *Salmonella* spp. in the Korean poultry production food supply chain is concerning due to the potential transmission to humans. High resistance to antimicrobials and multidrug resistance of *Salmonella* spp. from poultry sources were also reported in other countries [29,30]. For example, resistance to erythromycin, penicillin, and vancomycin have been reported as the most common resistance profile of *Salmonella* spp. from raw chicken meat in Malaysia [30] and poultry and poultry environments in India [31]. In Zhou *et al*.’s study [32], among 146 SEisolates obtained from retail chicken products in Shanghai, China, 50.70% of the isolates were resistant to ampicillin, 49.32% to sulfisoxazole, 17.12% to tetracycline, and 15.75% to doxycycline, and 30 (20.55%) isolates were resistant to three or more antimicrobials. In Canada, more than 43% of 193 *Salmonella* isolates recovered from commercial farms were simultaneously resistant to ampicillin, amoxicillin–clavulanic acid, ceftiofur, cefoxitin, and ceftriaxone [33].

A total of 90 SE isolates (93.8%) from different poultry sources showed the same virulence gene profile involved in adhesion, invasion, cell survival, and virulence plasmid genes. However, neither enterotoxin-producing genes nor biofilm regulator genes were detected in the strains tested. In previous studies [13,17,34], *Salmonella* isolates recovered from the animal, food, and/or environmental sources contained the same virulence genes as carried by human clinical isolates, and these findings highlight the pathogenic potential of *Salmonella* causing disease in humans via contaminated food.

Most SE isolates from the same origin phylogenetically clustered together even though sampling dates and sampling locations of the samples were different in this study. For example, the SE isolates from the retail chicken meats of brand A collected in April, July and August in 2011 clustered together in the group VII, and the SE isolates from the evisceration rooms, chilling rooms, and carcasses in slaughterhouse C all clustered together in the group IV (Supplementary Table S1, Figure 1 and Figure 2). Together, these data suggest the presence of a resident SE within the processing plant and slaughterhouse environments.

SE isolates from the different stages in the integrated broiler supply chain, such as grandparent stocks, parent stocks, hatcheries, and farms clustered together in the group X (Supplementary Table S1, Figure 1 and Figure 2). Interestingly, these SE isolates were collected in 2011 and 2017, and SE was not detected in the integrated broiler supply chain between 2012 and 2016 (data not shown). It remains uncertain how this SE strain was undetected and perpetuated in the chain during 2012–2016 and reappeared in 2017. Our SNP analysis data suggest that the SE strain persisted in the integrated broiler supply chain without an introduction of a new SE strain and evolved at a low nucleotide substitution rate of approximately 0.33 nucleotides per year compared to the evolutionary rate (1.01 nucleotide substitutions per year) estimated by a previous study of S. *enterica* [5]. Taken together, we assume that the SE strain was maintained at a low level in the integrated broiler supply chain but was not re-isolated until it reemerged in 2017.

The SE isolates from different origins showed close genetic relationships, and epidemiological or traceback evidence was provided to establish the connection between these SE isolates in the identical groups. In group IX, two SE isolates (KR 97 and KR 101) collected in the same year, 2012, from the slaughterhouse G and the truck which transported culled chickens to the slaughterhouse G were grouped together (Supplementary Table S1, Figure 1 and Figure 2). Therefore, the hypothesis that these isolates originated from the same source is supported by the epidemiological evidence and the phylogenetic analysis results.

Consistent with previous studies [17,19], both SNP and cgMLST analysis were highly congruent, but the SNP analysis provided a higher resolution than the cgMLST analysis in the phylogenetic analysis. The differences between SNP and cgMLST analysis may be due to a combination of these factors, i) a cgMLST scheme do not include intergenic regions, ii) only one allelic change are counted when multiple nucleotide changes within the same gene can be found, and iii) short insertions or deletions in the core genes are ignored by the SNP analysis, but captured by cgMLST analysis [17]. In addition, unlike an SNP analysis by the CFSAN SNP pipeline, no guideline for interpretation of the cgMLST results has been established; how many allelic differences are necessary to conclude that two strains are from the different origin [19].

## 4. Materials and Methods

### 4.1. Bacterial Isolates

The 96 SE isolates sequenced for this study were selected from SE isolates collected from poultry sources in South Korea between 2010 and 2017. The 83 isolates were isolated by Konkuk University in Seoul, South Korea, during 2010–2017, and 13 isolates were kindly provided by the Korean National Animal and Plant Quarantine Agency (APQA) in Gimcheon-si, South Korea (Supplementary Table S1).

For *Salmonella* isolation, 200 mL of buffered peptone water (BPW) (Difco Laboratories, Detroit, MI, USA) was added to the swab samples in sterile plastic jars with caps and incubated at 37 °C for 18–24 h. For the carcass rinse samples, rinsed BPW was transferred to a sterile plastic jar with a cap and incubated at 37 °C for 18–24 h for pre-enrichment. For selective enrichment, 100 µL of enriched BPW broth culture was transferred to 9.9 mL of Rappaport–Vassiliadis broth (Difco Laboratories) and incubated at 42 °C for 24–48 h. Following incubation, the Rappaport–Vassiliadis broth enrichment cultures were streaked onto xylose lysine deoxycholate agar (Difco Laboratories) and brilliant green agar (Difco Laboratories), and the plates were incubated overnight at 37 °C. Two presumptive *Salmonella* colonies from each sample were transferred to MacConkey agar (Difco Laboratories). Colonies with a positive result were confirmed by *Salmonella*-specific PCR, as previously described [35]. Serotyping was performed using antisera (Difco Laboratories) in agglutination tests on the basis of somatic O antigen and phase 1 and phase 2 flagella antigens according to the Kauffmann-White scheme.

Those isolates included 28 isolates from retail chicken meats of five different brands (A to E), 17 isolates from six chicken slaughterhouses (A to G), four isolates from two duck slaughterhouses, 37 isolates from hatcheries and chicken farms, two isolates from duck farms, and eight isolates from chicken transporting trucks. Among 96 SE isolates, 26 SE isolates were from the integrated broiler supply chain A; two grandparent stocks (*n* = 19), two hatcheries (*n* = 2), two parent stocks (*n* = 2), and two broiler farms (*n* = 3).

The slaughterhouse isolates originated from carcasses and environmental samples (chiller water and wall and floor of the bleeding room, evisceration room, chilling room, selection room, and processing rooms). The hatchery and farm isolates were from environmental samples (litter, dust, and drag swabs), carcasses, livers, feces, cloaca swabs, eggshells, and fluff.

### 4.2. Genome Sequencing

Sample DNA concentrations were determined using a Qubit BR dsDNA assay kit (Thermo Fisher Scientific Inc., Waltham, MA, USA). Concentrations were diluted to 0.2 ng/µL, and libraries were prepared following the Illumina Nextera XT DNA library prep kit (Illumina, Inc., San diego, CA, USA) reference guide with the following exceptions. Forty microliters of PCR product transferred to a new MIDI plate, 20 µL of AMPure XP beads (Beckman Coulter, Brea, CA, USA) were added to each well and incubated at room temperature for 5 min without shaking. After 80% ethanol washes, beads were allowed to air dry for 12 min. Then beads were resuspended in 53 µL of RSB and incubated at room temperature for 2 min without shaking.

The concentration of sample libraries was determined using the Qubit dsDNA HS assay kit (Thermo Fisher Scientific Inc., Waltham, MA, USA), and libraries were diluted to a 2 nM concentration and combined in equal volumes to form the pooled library. The pooled library was denatured to obtain a 10 pM final library following the Illumina Denature and Dilute Libraries Guide-Protocol A. Six hundred µl of the denatured 10 pM library was loaded into the MiSeq reagent cartridge.

### 4.3. Sequencing Reads Trimming, Filtering, and Classification

Trimmomatic [36] was used to remove low-quality reads. The leading three and the trailing three nucleotides were removed from the reads, and a 4-nucleotide sliding window was used to remove nucleotides from the 3′ end when the average Phred score dropped below 20. Additionally, reads shorter than 50 bp were discarded after trimming.

### 4.4. De Novo Assembly and Serotyping

The trimmed reads were assembled using SPAdes [34] with the “--careful” option. QUAST [37] was used to evaluate the quality of draft genome assemblies and determine the N50 value for each assembly. SeqSero [38] was used to predict *Salmonella* serotype from raw sequencing reads.

### 4.5. Antibiotic Resistance Analysis

The presence of acquired antimicrobial resistance genes as well as chromosomal mutations in the *gyrA*, *gyrB*, *parC*, and *parE* genes were determined using assembled genomes and ResFinder 3.2 (Center for Genomic Epidemiology, https://cge.cbs.dtu.dk/services/ResFinder/) with settings of a threshold of 90%, and a minimum length of 60% [39].

### 4.6. Virulence Genes Investigation

All genomes were annotated using the RASTtk algorithm [40] using the PATRIC v3.6.8. annotation server with default parameters (https://patricbrc.org/app/Annotation) [41]. Protein annotations, including those for proteins specifically asserted to be involved in virulence factors, were downloaded from the PATRIC workspace and used for subsequent analyses. Twenty-one virulence genes were investigated in this study, including adhesion effectors (*sef*, *pef*, *sfm and fim*), invasion effectors (*sop*, *inv.*, *org*, *sip*, *spa*, *sif*, *fli*, *flg*, *hil and spr*), host cell survival effectors (*ssa*, *sse*, *prg and pag*), virulence plasmid gene (*spv*), enterotoxin-producing gene (*stn*) and biofilm regulator (*bss*) [42].

### 4.7. Phylogenetic Analysis

High-quality SNPs were identified using CFSAN SNP Pipeline v2.0.2 using default quality filters [43]. Specifically, minimum base quality was 15; minimum mapping quality was 30; minimum fraction of reads for SNP calls was 0.9. Genomes of SE strain P125109 (NCBI reference sequence NC_011294.1) were used as reference genomes for reads mapping. Default pipeline settings were used for MiSeq reads. An SNP matrix and aligned SNPs were produced by the CFSAN SNP pipeline. Finally, maximum-likelihood (ML) phylogenetic trees were constructed using PhyML [44]. In addition, the phylogenetic data were visualized with antibiotic resistance patterns and virulence gene profiles using interactive Tree Of Life version 5 (iTOLv5) (https://itol.embl.de/).

For cgMLST, raw sequence data files of the isolates were uploaded to cgMLST Finder 1.1 of the Center for Genomic Epidemiology (https://cge.cbs.dtu.dk/services/cgMLSTFinder/) using the scheme developed by EnteroBase *Salmonella* database (https://enterobase.warwick.aC.uk) that considers a total of 3002 loci. The minimum spanning tree based on allelic profiles was created using GrapeTree version 1.5.0 [45].

## 5. Conclusions

In conclusion, this study demonstrated the application of WGS to predict antimicrobial resistance, detect virulence genes, and assess the phylogenetic relationship among SE isolates from different poultry sources in South Korea. In addition, both SNP and cgMLST analyses were highly congruent. Our results demonstrated that WGS is a powerful tool for investigating the epidemiology of *Salmonella* in the poultry industry, from farm to retail chicken meat, in conjunction with epidemiology and traceback findings. In addition, this study could be valuable reference data for future traceback investigations in the poultry industry in South Korea.

## Figures and Tables

**Table 1:** Antimicrobial genes detected in the 96 *Salmonella enterica* subsp. *enterica* serotype enteritidis (SE) isolates in this study.

Pattern	Antimicrobial Genes (Identity, %)						Chromosome Mutations	Number of Isolates (%)
	Aminoglycoside	Sulfonamide	Trimethoprim	Beta-Lactam	Tetracycline	Phenicol		
Pattern 1	*aac(6’)-Iaa* (96.4)*, aph(3’’)-Ib* (100), *aph(6)-Id* (99.76-100)	*sul2* (100)		*blaTEM-1B* (99.9-100)	*tet(A)* (99.9-100)		*gyrA* p.D87N	27 (28.1)
Pattern 2	*aac(6’)-Iaa* (96.4)*, aph(3’’)-Ib* (100), *aph(6)-Id* (99.76-100)	*sul2* (100)		*blaTEM-1B* (99.9-100)	*tet(A)* (99.9-100)		-	4 (4.2)
Pattern 3	*aac(6’)-Iaa* (96.4)*, aph(3’’)-Ib* (100), *aph(6)-Id* (100)	*sul2* (100)		*blaTEM-1B* (100)		catA2 (96.1)	*gyrA* p.D87G	5 (5.2)
Pattern 4	*aac(6’)-Iaa* (96.4)*, aph(3’’)-Ib* (100), *aph(6)-Id* (99.8)	*sul2* (100)		*blaTEM-1B* (100)			-	1 (1.0)
Pattern 5	*aac(3)-Iid* (99.9), *aac(6’)-Iaa* (96.4)*, aph(3’’)-Ib* (99.9)*, aph(3’)-Ia (100), aph(6)-Id* (100)	*sul2* (100)		*blaCTX-M-15* (100)	*tet(A)* (97.3-100)		*gyrA* p.D87N	15 (15.6)
Pattern 6	*aac(3)-Iid* (99.9), *aac(6’)-Iaa (96.4), aph(3’’)-Ib* (99.9)*, aph(3’)-Ia* (100)*, aph(6)-Id* (100)	*sul2* (100)		*blaCTX-M-15* (100)	*tet(A)* (97.3-100)		-	1 (1.0)
Pattern 7	*aac(6’)-Iaa* (96.4)	*sul2* (100)	*dfrA1* (95.8)	*blaTEM-1B* (100)			-	1 (1.0)
Pattern 8	*aac(6’)-Iaa* (96.4)	*Sul1* (100)	*dfrA1* (95.4)	*blaTEM-1B* (100)	*tet(A)* (98.8)		*gyrA* p.D87G	1 (1.0)
Pattern 9	*aac(6’)-Iaa* (96.4)						*gyrA* p.D87N	7 (7.3)
Pattern 10	*aac(6’)-Iaa* (96.4)						*gyrA* p.D87G	19 (19.8)
Pattern 11	*aac(6’)-Iaa* (96.4)						-	14 (14.6)
Pattern 12	*aac(3)-Iid* (99.9), *aac(6’)-Iaa (96.4)*			*blaCTX-M-15* (99.7)			*gyrA* p.D87N	1 (1.0)
Number of isolates (%)	96 (100)	55 (57.3)	2 (2.1)	56 (58.3)	48 (50%)	1 (1.0)	75 (78.1)	

**Table 2:** Virulence genes detected in the 96 SE isolates in this study.

Profile	Virulence Genes						Number of Isolates(%)
	Adhesion Effectors (*sef, pef, sfm and fim*)	Invasion Effectors (*sop, inv., org, sip, spa, sif, fli, flg, hil and spr*)	Host Cell Survival Effectors (*ssa, sse, prg and pag*)	Virulence Plasmid Gene (*spv*)	Enterotoxin-Producing Gene (*stn*)	Biofilm Regulator (*bss*)	
Profile 1	*Pef, fim*	*sop, inv., org, sip, spa, sif, fli, flg, hil*	*ssa, sse, prg, pag*	*spv*	-	-	90 (93.8)
Profile 2	*Fim*	*sop, inv., org, sip, spa, sif, fli, flg, hil*	*ssa, sse, prg, pag*	-	-	-	5 (5.2)
Profile 3	*Pef, fim*	*sop, inv., org, sip, spa, sif, fli, flg, hil*	*ssa, sse, prg, pag*	-	-	-	1 (1.0)

## Data Availability

The WGS data presented in this study are openly available in NCBI Bioproject database, Accession number (PRJNA623127).

## Supplementary Materials

The following are available online at https://www.mdpi.com/2076-0817/10/1/45/s1, Supplementary Table S1. Whole-genome sequencing characterization of 96 SE isolates used in this study.
