# Supplementary material for: Whole-Genome Sequencing Analysis of Salmonella Enterica Serotype Enteritidis Isolated from Poultry Sources in South Korea, 2010–2017
Source: Pathogens. 2021 Jan 7;10(1):45. doi: 10.3390/pathogens10010045 (PMC7825753; doi:10.3390/pathogens10010045)
Supplement: Supplementary file 1 [file pathogens-10-00045-s001.pdf]

Table 1. Whole-genome sequencing characterization of 96 SE isolates used in this study.

	Source	Sampling date (YYYY. MM)	Antimicrobial resistance pattern[a]	Chromosomal Mutations	Virulence gene profile[b]
KR1	Retail chicken meat (brand A)	2011.04	Pattern 5	gyrA p.D87N	Profile 1
KR2	Retail chicken meat (brand A)	2011.04	Pattern 5	gyrA p.D87N	Profile 1
KR3	Retail chicken meat (brand A)	2011.04	Pattern 5	gyrA p.D87N	Profile 1
KR4	Slaughterhouse A	2010.	Pattern 9	gyrA p.D87N	Profile 1
KR5	Retail chicken meat (brand A)	2011.05	Pattern 5	gyrA p.D87N	Profile 1
KR6	Retail chicken meat (brand A)	2011.06	Pattern 5	gyrA p.D87N	Profile 1
KR7	Retail chicken meat (brand A)	2011.07	Pattern 5	gyrA p.D87N	Profile 1
KR8	Retail chicken meat (brand A)	2011.07	Pattern 5	gyrA p.D87N	Profile 1
KR9	Retail chicken meat (brand A)	2011.07	Pattern 5	gyrA p.D87N	Profile 1
KR10	Retail chicken meat (brand A)	2011.08	Pattern 5	gyrA p.D87N	Profile 1
KR11	Retail chicken meat (brand A)	2011.08	Pattern 5	gyrA p.D87N	Profile 1
KR12	Retail chicken meat (brand A)	2011.08	Pattern 5	gyrA p.D87N	Profile 1
KR13	Retail chicken meat (brand B)	2011.02	Pattern 3	gyrA p.D87G	Profile 1
KR14	Retail chicken meat (brand B)	2011.03	Pattern 10	gyrA p.D87G	Profile 1
KR15	Retail chicken meat (brand B)	2011.03	Pattern 10	gyrA p.D87G	Profile 1
KR16	Retail chicken meat (brand B)	2011.03	Pattern 10	gyrA p.D87G	Profile 1
KR17	Retail chicken meat (brand B)	2011.04	Pattern 10	gyrA p.D87G	Profile 1
KR18	Retail chicken meat (brand B)	2011.04	Pattern 10	gyrA p.D87G	Profile 1
KR19	Retail chicken meat (brand C)	2011.11	Pattern 11	-	Profile 1
KR20	Retail chicken meat (brand D)	2011.02	Pattern 11	-	Profile 1
KR21	Retail chicken meat (brand D)	2011.02	Pattern 11	-	Profile 1
KR22	Retail chicken meat (brand D)	2011.03	Pattern 10	gyrA p.D87G	Profile 1
KR23	Retail chicken meat (brand D)	2011.03	Pattern 10	gyrA p.D87G	Profile 1
KR24	Retail chicken meat (brand D)	2011.03	Pattern 3	gyrA p.D87G	Profile 1
KR25	Retail chicken meat (brand D)	2011.05	Pattern 9	gyrA p.D87N	Profile 1
KR26	Retail chicken meat (brand D)	2011.06	Pattern 9	gyrA p.D87N	Profile 1
KR27	Retail chicken meat (brand D)	2011.09	Pattern 3	gyrA p.D87G	Profile 1
KR28	Retail chicken meat (brand D)	2011.09	Pattern 3	gyrA p.D87G	Profile 1
KR29	Retail chicken meat (brand E)	2011.03	Pattern 9	gyrA p.D87N	Profile 1
KR30	Slaughterhouse B	2011	Pattern 10	gyrA p.D87G	Profile 1
KR31	Slaughterhouse B	2011	Pattern 10	gyrA p.D87G	Profile 1
KR32	Slaughterhouse B	2011	Pattern 11	-	Profile 2
KR33	Slaughterhouse B	2011	Pattern 10	gyrA p.D87G	Profile 1
KR34	Slaughterhouse B	2011	Pattern 10	gyrA p.D87G	Profile 1
KR36	Slaughterhouse C	2011	Pattern 11	-	Profile 2
KR39	Slaughterhouse D	2011	Pattern 10	gyrA p.D87G	Profile 1
KR40	Duck Slaughterhouse A	2011.07	Pattern 7	-	Profile 1
KR41	Duck Slaughterhouse B	2011.07	Pattern 10	gyrA p.D87G	Profile 2

KR43	Duck Slaughterhouse B	2011.07	Pattern 11	-	Profile 1
KR44	Slaughterhouse E	2010	Pattern 11	-	Profile 1
KR45	Slaughterhouse E	2010	Pattern 9	gyrA p.D87N	Profile 1
KR46	Slaughterhouse F	2010	Pattern 10	gyrA p.D87G	Profile 3
KR47	Slaughterhouse F	2010	Pattern 10	gyrA p.D87G	Profile 1
KR48	Slaughterhouse F	2010	Pattern 10	gyrA p.D87G	Profile 1
KR49	Slaughterhouse F	2010	Pattern 10	gyrA p.D87G	Profile 1
KR50	Slaughterhouse F	2010	Pattern 10	gyrA p.D87G	Profile 1
KR51	Truck	2010.10	Pattern 9	gyrA p.D87N	Profile 1
KR53	Slaughterhouse G	2011.04	Pattern 9	gyrA p.D87N	Profile 1
KR54	Hatchery 1 (integrated broiler supply chain)	2011.05	Pattern 11	-	Profile 1
KR55	Farm 1 (integrated broiler supply chain)	2011.05	Pattern 1	gyrA p.D87N	Profile 1
KR56	Farm 2 (integrated broiler supply chain)	2011.05	Pattern 1	gyrA p.D87N	Profile 1
KR57	Grandparent stock 1 (integrated broiler supply chain)	2017.05	Pattern 1	gyrA p.D87N	Profile 1
KR58	Grandparent stock 1 (integrated broiler supply chain)	2017.05	Pattern 1	gyrA p.D87N	Profile 1
KR59	Grandparent stock 2 (integrated broiler supply chain)	2017.05	Pattern 1	gyrA p.D87N	Profile 1
KR60	Grandparent stock 2 (integrated broiler supply chain)	2017.05	Pattern 1	gyrA p.D87N	Profile 1
KR61	Grandparent stock 2 (integrated broiler supply chain)	2017.05	Pattern 1	gyrA p.D87N	Profile 1
KR62	Grandparent stock 2 (integrated broiler supply chain)	2017.05	Pattern 1	gyrA p.D87N	Profile 1
KR63	Grandparent stock 2 (integrated broiler supply chain)	2017.05	Pattern 1	gyrA p.D87N	Profile 1
KR64	Grandparent stock 2 (integrated broiler supply chain)	2017.05	Pattern 1	gyrA p.D87N	Profile 1
KR65	Grandparent stock 2 (integrated broiler supply chain)	2017.05	Pattern 1	gyrA p.D87N	Profile 1
KR66	Grandparent stock 1 (integrated broiler supply chain)	2017.05	Pattern 1	gyrA p.D87N	Profile 1
KR67	Grandparent stock 2 (integrated broiler supply chain)	2017.05	Pattern 1	gyrA p.D87N	Profile 1
KR68	Grandparent stock 2 (integrated broiler supply chain)	2017.05	Pattern 1	gyrA p.D87N	Profile 1
KR69	Grandparent stock 2 (integrated broiler supply chain)	2017.05	Pattern 1	gyrA p.D87N	Profile 1
KR70	Grandparent stock 2 (integrated broiler supply chain)	2017.05	Pattern 1	gyrA p.D87N	Profile 1

KR71	Grandparent stock 2 (integrated broiler supply chain)	2017.05	Pattern 1	gyrA p.D87N	Profile 1
KR73	Grandparent stock 2 (integrated broiler supply chain)	2017.05	Pattern 1	gyrA p.D87N	Profile 1
KR74	Grandparent stock 2 (integrated broiler supply chain)	2017.05	Pattern 1	gyrA p.D87N	Profile 1
KR75	Grandparent stock 2 (integrated broiler supply chain)	2017.05	Pattern 1	gyrA p.D87N	Profile 1
KR76	Grandparent stock 2 (integrated broiler supply chain)	2017.05	Pattern 1	gyrA p.D87N	Profile 1
KR77	Hatchery2 (integrated broiler supply chain)	2017.05	Pattern 1	gyrA p.D87N	Profile 1
KR78	Farm 3 (integrated broiler supply chain)	2017.07	Pattern 1	gyrA p.D87N	Profile 1
KR79	Parent stock 1 (integrated broiler supply chain)	2017.06	Pattern 1	gyrA p.D87N	Profile 2
KR81	Chicken farm_KNAPQA[c]	2013	Pattern 11	-	Profile 1
KR82	Chicken farm_KNAPQA	2013	Pattern 11	-	Profile 1
KR83	Chicken farm_KNAPQA	2014	Pattern 10	gyrA p.D87G	Profile 1
KR84	Duck farm_KNAPQA	2014	Pattern 1	gyrA p.D87N	Profile 1
KR85	Chicken farm_KNAPQA	2014	Pattern 1	gyrA p.D87N	Profile 2
KR86	Chicken farm_KNAPQA	2015	Pattern 5	gyrA p.D87N	Profile 1
KR87	Chicken farm_KNAPQA	2015	Pattern 11	-	Profile 1
KR88	Hatchery_KNAPQA	2016	Pattern 2	-	Profile 1
KR90	Duck farm_KNAPQA	2016	Pattern 2	-	Profile 1
KR91	Chicken farm_KNAPQA	2017	Pattern 2	-	Profile 1
KR92	Chicken farm_KNAPQA	2017	Pattern 2	-	Profile 1
KR93	Chicken farm_KNAPQA	2017	Pattern 11	-	Profile 1
KR94	Chicken farm_KNAPQA	2017.05	Pattern 11	-	Profile 1
KR95	Parent stock 2 (integrated broiler supply chain)	2017.05	Pattern 1	gyrA p.D87N	Profile 1
KR96	Slaughterhouse G	2012.03	Pattern 3	gyrA p.D87G	Profile 1
KR97	Slaughterhouse G	2012.03	Pattern 4	-	Profile 1
KR99	Truck	2012.04	Pattern 5	gyrA p.D87N	Profile 1
KR100	Truck	2012.04	Pattern 12	gyrA p.D87N	Profile 1
KR101	Truck	2012.04	Pattern 11	-	Profile 1
KR102	Truck	2012.04	Pattern 5	gyrA p.D87N	Profile 1
KR103	Truck	2012.04	Pattern 6	-	Profile 1
KR104	Truck	2012.04	Pattern 5	gyrA p.D87N	Profile 1
KR105	Truck	2012.06	Pattern 8	gyrA p.D87G	Profile 1

[a] Antimicrobial resistance patterns are shown in Table 1.

[b] Virulence gene profiles are shown in Table 2.

[c] KNAPQA; Korean National Animal and Plant Quarantine Agency
